# Iron-Oxide Nanoparticles Embedded in 3D-Printed PLA/HA Scaffolds for Magnetic Hyperthermia Therapy: An Experimental–Numerical Analysis of Thermal Behavior

**DOI:** 10.3390/ma17235836

**Published:** 2024-11-28

**Authors:** Serxio Álvarez-Olcina, Miriam López-Álvarez, Julia Serra, Pío González

**Affiliations:** 1 Grupo Novos Materiais, CINTECX (Centro de Investigación en Tecnoloxía, Enerxía e Procesos Industriais), Universidade de Vigo, 36310 Vigo, Spain; sealvarez@alumnado.uvigo.gal (S.Á.-O.); jserra@uvigo.gal (J.S.); pglez@uvigo.gal (P.G.); 2 IISGS (Instituto de Investigación Sanitaria Galicia Sur), Servicio Galego de Saúde-Universidade de Vigo (SERGAS-UVIGO), 36213 Vigo, Spain

**Keywords:** magnetic hyperthermia, iron-oxide nanoparticles, 3D-printed scaffolds, polylactic acid

## Abstract

Hyperthermia is nowadays intensively investigated as a promising strategy to improve the therapeutic efficacy against different types of cancer and resistant infections. In particular, the remote generation of localized hyperthermia by magnetic field through iron-oxide nanoparticles (IONPs) offers good thermal conductivity in a controlled area. The incorporation of these IONPs in 3D-printed scaffolds designed for bone tissue regeneration has been scarcely addressed in the literature. This strategy would add the potential of magnetic-mediated hyperthermia against remnant cancer or resistant infections in the damaged tissue area to these personalized bone-related scaffolds. The present work proposes two methodologies to obtain 3D-printed bone-related scaffolds with magnetic properties: 1-Direct 3D printing with IONPs-embedded polylactic acid (PLA) and hydroxyapatite (HA), resulting in a uniform distribution of IONPs; and 2-Drop coating on 3D-printed PLA/HA scaffolds, resulting in the IONPs being concentrated on the scaffold surface. Physicochemical/mechanical characterizations were performed to confirm the IONPs’ distributions and viability assays were carried out to validate the absence of cytotoxicity. Hyperthermia tests (314 kHz) were carried out, including the simulation/validation of the experimental equipment, to establish optimal distances from the planar coil. Temperature–time/distance curves were obtained and parametrized (R^2^ > 0.96) for both methodologies in relation to the contribution of IONPs (0.20–1.00 mg), their distribution in the scaffold (uniform/concentrated), the electric-current intensity, and the distance. The results validated both methodologies to obtain personalized 3D-printed PLA/HA scaffolds with magnetic properties, reaching the required moderate/ablative hyperthermia levels.

## 1. Introduction

Local hyperthermia therapy has recently become one of the most intensively explored strategies, especially for the treatment of different types of cancer and drug-resistant bacterial infections [1], which are two of the main health challenges worldwide [2,3,4,5,6]. In particular, the administration of local moderate hyperthermia (43–50 °C) has been proven to selectively inhibit the growth and promote the apoptosis of cancer cells, instead of the undesired necrosis, as the healthy tissue is able to resist, due to basic physiological differences between both tissue vasculatures [7,8]. Recent studies have shown that when hyperthermia is used as adjunct therapy along with chemotherapy or radiotherapy, the efficacy of the treatment increases without considerably increasing toxicity in healthy tissues [2,9]. In the case of a strategy against bacterial infections, certain doses of moderate and ablative (50–55 °C) hyperthermia have been proven, alone or in combination with antibiotics or antimicrobial peptides, to inhibit the growth of drug-resistant bacterial biofilms, affecting their motility and cell-wall integrity [1,10,11,12]. In this sense, accurate local application of the required levels of hyperthermia must be ensured with thermal doses specifically determined for each case, limiting the area of action of the treatment to the damaged tissues and guaranteeing a minimal impact on the surrounding healthy tissues [1,4].

Several technological approaches have been developed for applying local hyperthermia without causing damage to the surrounding tissue, such as radiofrequency ablation, focused ultrasound, laser ablation, and magnetic-mediated hyperthermia (MMH) [13,14]. Among them, MMH stands out because of its high efficiency in providing the temperature rise. This hyperthermia is achieved under an external alternating magnetic field (AMF) generator, which induces electrical currents on magnetic nanoparticles situated inside the body in close contact with the targeted region, resulting in the generation of heat [2,13]. The use of nanoparticles facilitates locally controlled applications and provides unique properties owing to the high fraction of surface atoms and surface energy, as well as the quantum effects produced by their spatial confinement. Moreover, the large surface-to-volume ratio and small size facilitate their distribution over many binding sites as well as their ability to deeply penetrate tumor tissues [15]. This strategy provides other advantages, such as the absence of penetration depth limitations or complex wave distortions through tissue boundaries [16]. The key parameters to be controlled for efficient heating in the MMH are the magnetic nanoparticle properties, size, and materials, and the magnetic field parameters (amplitude and frequency) [2].

Among the magnetic nanoparticles, those composed of magnetite (Fe_3_O_4_) or iron oxide (II, III), known as IONPs, have been the most investigated. These nanoparticles can be easily synthesized and functionalized to increase their internalization by target cells. In fact, magnetite-based hyperthermia has already been applied to patients in controlled studies, given its low cytotoxicity and tunable magnetic properties that promote the warming of the cells closest to the IONPs with minimal effect on the surrounding healthy tissue [15]. In this sense, these IONPs can be incorporated directly into the living tissues of interest but can also be combined with other biomaterials to develop multifunctional devices. For instance, the incorporation of these IONPs on biocompatible and biodegradable scaffolds, designed for bone tissue regeneration purposes, would add the potential of magnetic-mediated hyperthermia against remnant cancer or resistant infections in the damaged tissue area to scaffolds. Currently, research on bone tissue engineering has been intensified towards the obtaining of 3D-printed scaffolds, by additive manufacturing, based on biodegradable polymers such as polylactic acid (PLA), to provide personalized scaffolds with complex morphologies. Three-dimensional printing technology also allows for direct fabrication using an in situ prepared composite mixture combining polylactic acid with other components of interest, such as hydroxyapatite (HA) in bone-related applications [17]. In this sense, the incorporation of these IONPs in scaffolds obtained by 3D-printing methodologies has been scarcely addressed in the literature [18]. Several works presented the incorporation of IONPs into PLA scaffolds by using additive fabrication methodologies, such as selective laser sintering, to improve mechanical properties and cell adhesion [19]. This methodology offers high accuracy. However, it requires high-powered lasers, which increase the cost and the safety requirements. The incorporation of IONPs by 3D printing, known as fused deposition modeling, was recently addressed [20] in porous calcium silicate/polycaprolactone (PCL) scaffolds. The authors proved that the presence of IONPs together with daily magnetic stimulation increased the differentiation of mesenchymal stem cells to bone regeneration. To fabricate these scaffolds, a paste was previously prepared, requiring previous procedures at high-temperature furnaces and ball milling, before being introduced into the 3D-printing cartridge. Another recent approach [21] proposes a magnetic 3D-printed scaffold combining PLA with HA, IONPs, and antibiotics against bacterial biofilm and the re-growing of bone tissue. The procedure involves several steps, and the first one is the 3D printing of a PLA filament, continuing with alkaline hydrolysis, washing, and soaking processes (24 h) to load it with nanosized HA, IONPs, and antibiotics.

The present work proposes two easy-to-implement and cost-effective methodologies for the incorporation of IONPs at different concentrations (from 0.10 to 0.50 wt.%) in 3D-printed PLA/HA scaffolds and their thermal behavior evaluation for local magnetic moderate/ablative hyperthermia levels, required against remnant cancer of resistant infections. The methodologies proposed are as follows: (1) The 3D printing of IONPs directly incorporated with the in situ mixture of PLA and HA, generating a uniform distribution inside the scaffold, and (2) the drop coating of the IONPs on the 3D-printed 3D PLA/HA scaffolds, with the IONPs concentrated on one surface of the scaffold. Physicochemical (optical microscopy, SEM/EDS, FT-Raman) and mechanical characterizations (compression tests) of the scaffolds with different IONP concentrations by the two methodologies are presented to confirm the presence of IONPs and evaluate their distribution and influence. Then, a simulation and experimental validation of the induced AMF provided by the planar coil (314 kHz) were carried out to optimize the experimental conditions. The thermal behavior of the scaffolds obtained with different IONP concentrations and by the two proposed methodologies was then presented, and their ability to generate the required moderate (42–50 °C) or ablative (50–55 °C) hyperthermia assessed for AMF exposition times of 120 s. The influence on the thermal behavior of parameters related to the AFM generator, such as the distance of the scaffolds from the planar coil, and the electric-current intensity applied (varied from 90 to 190 A), were also studied, together with the influence of the individual parameters related to the scaffold, such as the IONPs’ concentrations and the distribution of these IONPs (uniformly, 3D-printed or concentrated on the external layer, drop coating). Finally, preliminary viability assays with the MG63 cell line were performed to validate the absence of potential toxins released from the scaffolds.

The Materials and Methods section describes in detail the fabrication methodologies, the compositional contributions of PLA, HA, and IONPs for each methodology, the printer parameters, and the techniques/procedures used for the physicochemical, mechanical, and biological characterization of the scaffolds. The equipment used to generate the alternating magnetic field (AMF) is also detailed in the Materials and Methods section, together with a simulation (and experimental validation) of the 3D distribution of the AMF generated by the setup used, to elucidate the more efficient position in the X-Y plane and the Z-axis to situate the scaffold, in relation to the planar coil. Then, the AMF experimental methodology carried out, situating the scaffolds on the already defined position, is also described. The Results section incorporates the physicochemical, mechanical, and biological characterizations, and the corresponding temperature–time measurements obtained after 120 s of AMF irradiation for both types of scaffolds, varying the intensity (90–190 A) and the IONP contributions (0.20 to 1.00 mg/scaffold).

## 2. Materials and Methods

### 2.1. Raw Materials

Natural polylactic acid (PLA) SMARTFIL^®^ pellets with an oval shape and dimensions of 5.0 mm and 3.5 mm for the major and minor axis, respectively, were purchased from Smart Materials (Jaén, Spain). Their primary properties are listed in Table 1. Hydroxyapatite (HA) Captal^®^ ‘R’ (batch P120R) powder with a spherical morphology (average particle size 3.29 µm) was acquired from Plasma-Biotal Limited (Buxton, UK). According to the manufacturers, it presents a Ca:P ratio in the range 1.66–1.72, a crystallinity around 85–95% and a high surface area of typically 6–20 m^2^/g. Iron-oxide (III) (Fe_3_O_4_) nanoparticles (IONPs) with an average particle size of 15 nm in an aqueous suspension of 5 mg/mL were purchased from Sigma-Aldrich (product. number 900043, Merck, Madrid, Spain).

### 2.2. Scaffolds Fabrication: 3D-Printing and Drop-Coating Procedures

Two methodologies were used to obtain the intended scaffolds. The first implies direct 3D printing for a mixture of the three compounds. For this, the corresponding amount of PLA was first carefully and repeatedly mixed in a Petri dish using a spatula with HA powder in a 5 wt.% of the total mass and different contributions of IONPs: 0.10, 0.20, and 0.50 wt.%. Mixtures free of IONPs (0.00 wt.%) and PLA alone were also prepared to obtain control scaffolds. The contributions of PLA, HA, and IONPs incorporated into the mixtures to the scaffolds and controls are summarized in Table 2. Once obtained, each single mass was introduced in the 3D-FDM printer hopper (TUMAKER NX Pellet, Irun, Spain) to obtain the 3D-printed PLA/HA scaffolds shaped as disks with dimensions of 8 mm in diameter and 2.5 mm in height (0.1 mm height per layer) with 100% on infill density conditions. The scaffolds were first designed using SolidWorks 2016 software, and the digital data from the designs were then saved as STL files to generate the corresponding G-code sets for 3D printing using the Simplify3D software (4.1.2). The bed temperature of the 3D-FDM printer was maintained at 50 °C using a nozzle with a filament of 0.8 mm and an infill with a rectilinear pattern of an alternating 90° angle from one layer to the overlapped one. The printer had two extruders and two-point temperature controls, which were adjusted to *T*_1_ = 260–265 °C and *T*_2_ = 185–190 °C to favor the printing of the different mixtures, together with the flow rates. The control scaffolds were fabricated at lower temperatures and higher flow rates. The printing parameters used are listed in Table 3.

**Figure 1:**
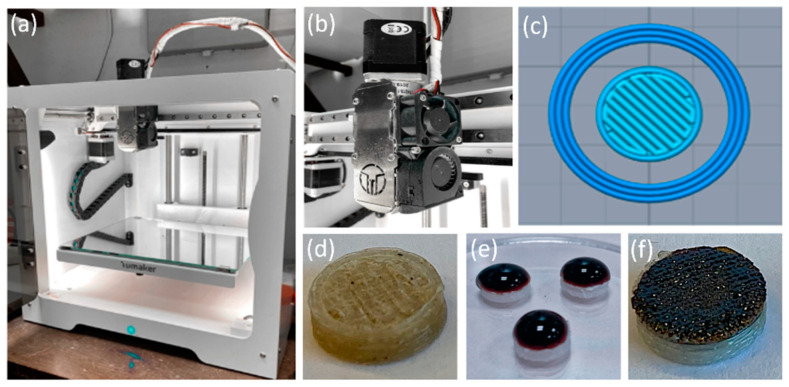
3D-FDM printer (**a**); extruder in detail (**b**); the samples’ design from the Simplify3D Professional Software (**c**); a sample of the scaffolds obtained by the 3D direct printing methodology (**d**) and by the drop coating of the IONPs on the already printed 3D PLA/HA scaffolds (**e**,**f**).

The other set of scaffolds was fabricated using a combination of 3D printing and drop coating. The drop-coating technique, in comparison to other techniques such as the use of a combined filament in 3D printing, allows for a better understanding of the heating process, because the generation of heat is precisely located on the coated surface. Using the 3D-printed PLA/HA scaffolds as the base material (95:5 wt.%), a drop of an aqueous suspension of IONPs was placed on top of the upper surface of the scaffolds. To obtain different deposited masses (0.25, 0.50 and 1.00 mg/scaffold), 50 µL of the 5 mg/mL IONP suspension was pipetted onto the upper face of the PLA/HA disks at the required times, followed by a drying process in a laboratory oven at 37 °C for 24 h after each drop deposition. A volume of 50 µL was selected to ensure complete coating of the PLA/HA disk surface (with a nominal diameter of 8 mm). Table 4 summarizes the set of drop-coated scaffolds and their nomenclature. In Figure 1, the details of the 3D-FDM printer used are shown together with the Simplify3D Professional Software image of the design, along with the two methodologies followed to incorporate the IONPs into the PLA/HA scaffold: (1) 3D direct printing and (2) drop coating on the already 3D-printed PLA/HA scaffolds.

### 2.3. Physicochemical Characterization

The global structure of the scaffolds was first analyzed using a stereomicroscope (Nikon SMZ25, Tokyo, Japan), and the surface morphology was analyzed using a scanning electron microscope (SEM; FEI Quanta 200 high-resolution, CACTI, University of Vigo, Vigo, Spain). The samples were first mounted on metal stubs and sputter coated with carbon. The elemental composition was determined by EDS using an Oxford Inca Energy 300 coupled to an SEM microscope (Buckinghamshire, UK). Moreover, FT-Raman spectroscopy was performed to identify the main molecular vibrations and corresponding functional groups using a B&W Tek i-Raman-785S instrument (Metrohm, Herisau, Switzerland) equipped with a BAC 100 probe (785 nm) in the wavenumber range of 250–3250 cm^−1^ with an incident laser radiation of 340 mW. Young’s moduli of the scaffolds were determined from stress–strain curves (previous conversion to engineering stress and engineering strain) under quasistatic unidirectional compression tests in a tensile bench with a 5 kg load cell (TA.XTPlusC, Texture Technologies Corp. and Stable Micro Systems, Ltd., Godalming, UK) at a crosshead speed of 0.01 mm/s. The compression tool comprised a stainless-steel cylinder with a diameter of 12.7 mm, applied on the top surface of the scaffolds with a maximum workload of 45 N. The results are expressed as the mean values of five measurements for each condition. The instantaneous elastic deformation was calculated from the ratio of the initial height to the height of the sample at each step of the compression test with a sampling frequency of 400 ppp.

### 2.4. AMF Hyperthermia: Equipment, Experimental Tests and Numerical Simulations

**Figure 2:**
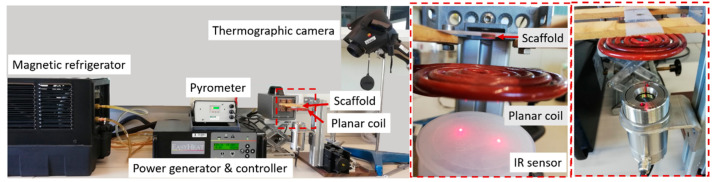
Alternating magnetic field generator and coupled used equipment.

An alternating magnetic field (AMF) generator was used, with a planar coil (five turns with an inner diameter of 10.5 mm and an outer diameter of 62.0 mm) driven by a transistor inverter (EasyHeat RHS 0224, Ambrell Corp., New York, NY, USA) operated at a frequency of 314 kHz and an electric current (EC) between 90 and 190 A. Moreover, the AMF generator and the coil were both cooled by a water-recirculating coolant system (FlowMax 230, MILLER Electric Mfg. Co., Appleton, WI, USA) with an average mass flow of 0.7 g/m^3^. The experimental setup is presented in Figure 2. The heating effect was measured by thermograph images using an infrared thermal-imaging camera (Testo 881, Testo Inc., Titisee-Neustadt, Germany) and a laser-guided infrared pyrometer (CTL, Micro-Epsilon GmbH & Co., Otenburg, Germany).

The setup of the experimental equipment included a simulation of the induced AMF using MATLAB software R2024A. The intention of this simulation is to visualize the distribution and magnitude of the AMF generated specifically in our experimental setup at any point in the space and, with this information, elucidate the most efficient position in the X-Y plane and the Z-axis to situate the magnetic 3D-printed scaffolds (also considering the specific scaffold’s dimensions, shaped as disks 8 mm in diameter, and 2.5 mm in height), in relation to the planar coil used. The experimental setup was modeled through the discretization of the five coils by concentric arrangements of 300 points of the same radius. Subsequently, the Biot–Savart law (an experimental physics law that relates the magnetic field to distance) was computed at each point for an equivalent current of 75 A flowing through the coils, which is a safe current value for the validation equipment. The space around the coils was also divided into a grid of 200 × 200 × 200 points, so that the intensity of the static magnetic field could be calculated for each of the points.

**Figure 3:**
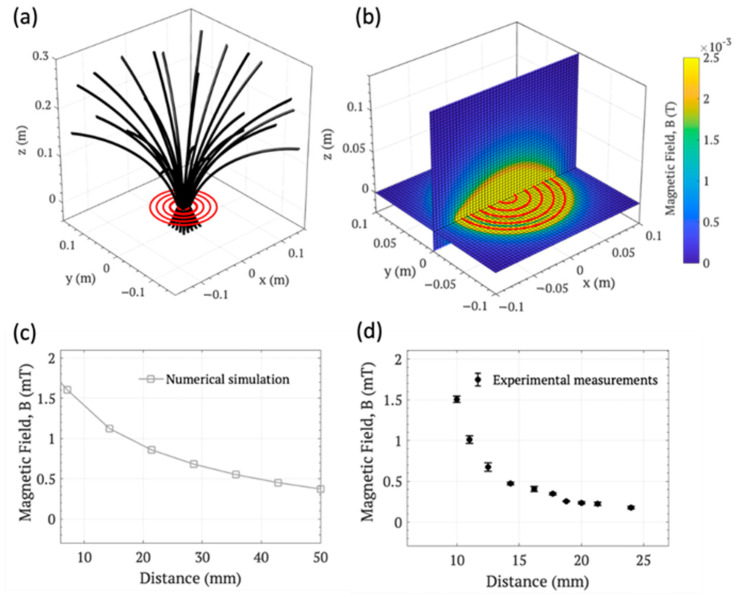
A graphical representation of the magnetic flux lines induced by the coils (**a**); a simulation of the AMF modulus distribution along the symmetrical planes of the coils (**b**); the results of the numerical simulation of the AMF modulus intensity (**c**) and experimental values measured (**d**).

The simulation results were validated by experimental data using a spectrum analyzer (Spectran^®^ NF-5030, Euscheid, Germany), which senses the real magnetic field intensity in the z-axis of the coil, confirming the same tendency provided by the simulation. The distribution of the AMF obtained, represented in Figure 3a, indicates that the closed field lines originate from the center point of the planar coil, together with the alternating polarity responsible for producing parasitic currents in the scaffolds and, consequently, remotely generating heat. Moreover, the AFM modulus (mT) simulation, represented in the color map (Figure 3b) over two perpendicular planes cutting the planar coil on its corresponding axes, clearly situates the maximum AFM modulus at the center point of the coil and in its vertical plane (Z-axis). Therefore, as expected, the scaffolds must be centered on the Z-axis. The maximum values of the AMF modulus obtained under the simulation along the Z-axis, represented in Figure 3c, against the distance from the center point of the planar coil, gradually decreased with distance, following a decreasing exponential curve. Based on this result, together with the dimensions of the scaffolds and to facilitate handling, the optimal distance for the experiment was set in the range from 10 mm to 24 mm from the planar coil in the z-axis, being preferentially situated at 10 mm or closer to maximize the AMF intensity. As observed in Figure 3d, experimental values obtained using an electromagnetic field spectrum analyzer followed the same tendency in a decreasing exponential curve (Figure 3d), but with lower values for the same distance. This can be easily attributed to the always present limitations in a simulation, to exactly reproduce the real conditions as well as the energy losses in the experimental equipment (heat, magnetic permeability, etc.) and the accuracy limitations of the measuring instrument.

With this information, experimental tests were then carried out with the 3D-printed scaffolds for the two proposed methodologies: the 3D-printed and drop-coated scaffold sets. First, temperature measurements were performed for each sample after 120 s of heating while varying the EC flowing through the induction coil and the distance between the surface of the scaffolds and the coil center. The values of the EC were selected to be 90 A, 120 A, and 190 A, while the measured distance from the coil ranged from 9.60 mm to 23.60 mm. A heating time (120 s) was selected to ensure that the thermal conditions reached a steady state and to measure the saturation temperature in each test. Thus, the dependence of heat generation on the IONP concentration and the alternating magnetic field (AMF) strength was assessed using temperature–distance curves obtained for the setup conditions and the composition of the scaffolds. Second, the induction heating process was evaluated within the first 120 s for all the samples, carrying out temperature measurements every 10 s at a fixed distance of 9.60 mm and an EC of 90 A. This approach allows for the representation of temperature–AMF exposure time curves that better describe the heating process of the scaffolds before reaching thermal stability. To obtain parametric equations that can model and predict the transient thermal behavior, the experimental data were fitted to the exponential equation Equation (1), frequently used to model the induction heating of IONPs in aqueous solutions [22]. Equation (1) is as follows:

(1) $$T=∆T\;(1-e^{-kt})$$

This equation is taken directly from Newton’s law of cooling, which describes the rate of heat loss of an object to its surroundings. It states that the rate of heat loss is directly proportional to the temperature difference between the object and its surroundings. The hotter an object is, the faster it will cool down. This law is equivalent for heating: the hotter an object is, the slower it will heat up, where k is a proportionality constant that depends on the characteristics of the object and environment. Moreover, this equation can be used to calculate the final saturation temperature, where t → ∞.

### 2.5. Biological Response In Vitro: Cytotoxicity Assay

Before the cell assays, a set of scaffolds obtained by the 3D-printing methodology was packed in a laminar flow cabin and sterilized with a direct source of UV light for 20 min on each side. To evaluate the cytotoxicity of the potential release of small particles from the scaffolds, a solvent extraction test [23] was performed. To obtain the extracts, the corresponding number of disks, with a ratio of 3 cm^2^ of material per ml of supplemented growth medium were placed in individual test tubes with the growth medium DMEM (Lonza) supplemented with 10% fetal bovine serum (HyClone) and 1% of a combination of penicillin, streptomycin, and amphotericin B (Lonza), and maintained in shaking conditions at 120 rpm for 24 h at 37 °C. A positive control for cytotoxicity (phenol solution in growth medium, 6.4 g/L) and a negative control for cytotoxicity (the culture medium itself) were also incubated under the same conditions. The negative control of cytotoxicity is defined [23] as a liquid medium/material proven to not promote any cytotoxic response, as it is the culture medium supplemented with serum. The positive control of cytotoxicity must be a solvent that promotes a reproducible cytotoxic response, mentioning phenol dilutions specifically as one of the possibilities. The purpose of the negative/positive controls is to demonstrate an appropriate test system response. Cells must behave properly, being viable when cultured with culture medium, and not viable when cultured with dilutions of phenol (6.4 mg/mL), to validate the experiment. After the extraction period, the complete volume of the extract (100%) and the controls subjected to the same conditions were recovered, and different dilutions were prepared (50, 30, 10, and 0%) in fresh supplemented culture medium.

A cell suspension (7 × 10^4^ cells/mL) of the human osteosarcoma cell line MG63 (ECACC 86051601) in growth medium was seeded in a 96-well microplate at a volume of 100 μL per well at 37 °C in a humidified atmosphere with 5% CO_2_. After 72 h of incubation, the medium of the subconfluent cell layer was replaced in each well with the previously prepared extracts. Four replicates per concentration were incubated with cells for 24 h. Cell viability was quantified using the MTS Cell Proliferation Assay Kit (Abcam, Cambridge, UK). This colorimetric assay is based on the MTS tetrazolium compound, which is exclusively reduced by viable cells to generate a colored formazan dye that is soluble in the culture medium. MTS reagent (10 μL) was then added to each well. After 45 min of incubation, the absorbance of the resulting solutions was measured at a wavelength of 490 nm using a microplate spectrophotometer (Bio-Rad, Hercules, CA, USA). Two independent experiments were performed. Wells with the corresponding extract solution without cells were also tested under the same conditions and subjected to the MTS colorimetric test as a blank control to ensure the avoidance of false positives.

### 2.6. Statistical Analysis

Data were analyzed using GraphPad Prism 10.3.1 (GraphPad Software Inc., San Diego, CA, USA), and the results are represented graphically as the mean ± standard deviation/standard error. The nonparametric Kruskal–Wallis test was used to determine the statistical differences, and a nonparametric Mann–Whitney test was used to perform the paired comparison. Statistical significance was determined to be * (*p* ≤ 0.05) at the 95% confidence level.

## 3. Results

### 3.1. Morphological and Physicochemical Characterization

**Figure 4:**
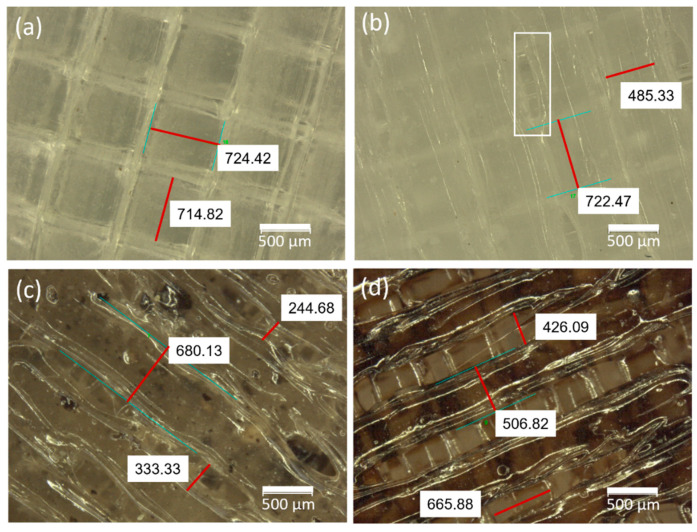
Stereomicroscope images of the 3D-printed scaffolds of PLA (**a**) and PLA/HA (3D0.00) (**b**) scaffolds obtained by 3D-printing methodology (**c**), specifically 3D0.40; and of the scaffolds obtained by the IONP drop coating on the 3D-printed PLA/HA scaffolds’ (**d**), the drop-coating methodology, specifically DC0.50.

Once the scaffolds were obtained by the following two methodologies: (1) the 3D printing of IONPs directly incorporated (0.20, 0.40, and 1.00 mg/scaffold) with the corresponding amounts of PLA and HA and (2) the drop coating of the IONPs (0.25, 0.50, and 1.00 mg/scaffold surface) on the already 3D-printed PLA/HA scaffolds; all the scaffolds were analyzed by stereomicroscopy. Moreover, 3D-printed scaffolds of PLA alone and of PLA/HA without IONPs (also named 3D0.00 and DC0.00) were also evaluated as reference materials. Figure 4 shows stereomicroscope images of the 3D-printed scaffolds of PLA alone (a), PLA/HA (b), and the 3D-printed scaffolds with IONPs directly incorporated (c); specifically, 3D0.40 is the one shown. The morphology of the IONPs drop-coated on the 3D-printed PLA/HA disks is also represented, specifically DC0.50, as shown in Figure 4d. The 3D-printed PLA scaffolds (a) clearly revealed that they followed a rectilinear printing pattern, alternating a 90° angle from one layer to the overlapped one, promoting a uniform square-shaped morphology on each layer (with side dimensions of 724.42 ± 0.01 µm) close to the nozzle diameter (0.8 mm) of the printer, as expected. When HA powder was incorporated (5 wt.%), 3D0.00, the 3D-printed PLA/HA scaffold (b) gained opacity, given the presence of HA. Moreover, it was observed that the square-shaped pattern became more irregular with filament diameters from 720 µm to narrower ones (red lines) of approximately 500 µm along the printing line, promoting the presence of oval pores (white rectangle). When IONPs were incorporated (c) (together with the HA and the PLA) by the 3D direct printing, the irregularity in the diameter of each deposited filament was intensified; in the case of 0.40 mg, this presented a change in the diameter of the filament in the range from around 700 µm to less than 300 µm. Moreover, the general view of the samples revealed a change in color to a darker color caused by the incorporation of IONPs. On the contrary, when IONPs were incorporated by drop coating (d), the pattern remained more regular in the same range of diameter for the deposited filament as in the PLA/HA scaffolds (b), as expected, with the IONPs concentrated in the upper layer and not uniformly distributed inside the filament as in the 3D-printed samples. According to the previous literature, it is thought that the variations in the 3D-printed filament thickness during material deposition are directly related to the printing conditions and rheology of the composite materials used [7].

**Figure 5:**
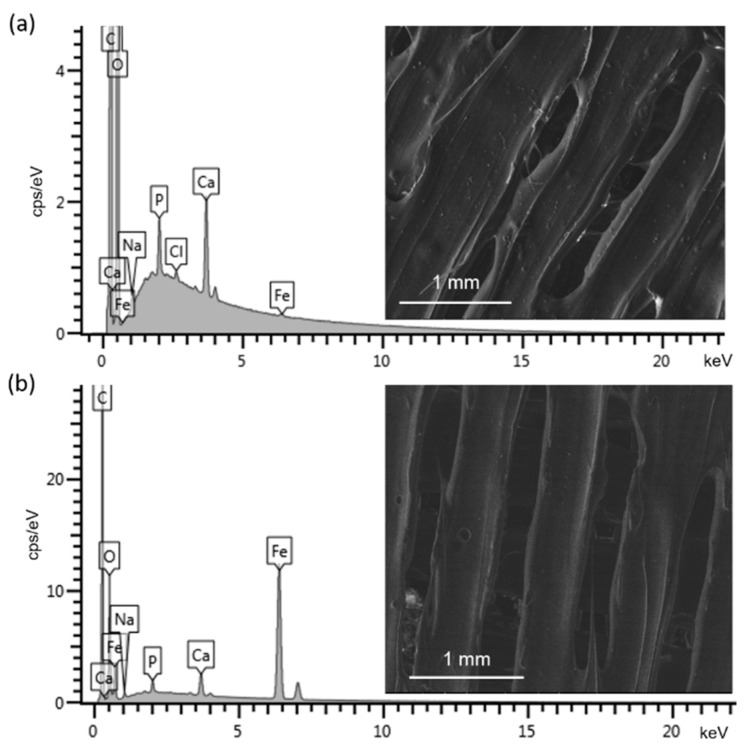
The SEM micrographs and corresponding EDS spectra of scaffolds fabricated with both methodologies: (1) the 3D printing, namely with the sample 3D0.40 (**a**); and (2) the drop coating, with the sample DC0.50 (**b**).

A more detailed analysis of the scaffold morphology obtained from both methodologies was carried out by SEM, along with the elemental composition by EDS, both of which are presented in Figure 5 for the respective 3D0.40 (a) and DC0.50 (b) scaffolds. First, in the SEM micrograph of 3D0.40 (a), irregularities on the edges and variations in the width or diameter along the printing lines, as already mentioned for the stereomicroscope analysis (Figure 4c), were clearly observed. For the drop-coated disks (DC0.50, b), a more uniform diameter along the printing lines (as previously shown in Figure 4d) was observed again. The corresponding EDS spectra (Figure 5) revealed the expected peaks of C in both cases, attributed to the presence of PLA and, to a high extent, the sputtering of the scaffolds with the same element to facilitate the SEM analysis. The O peak was also clearly detected in both scaffolds, as were the peaks from Ca and P, corresponding to the contribution of hydroxyapatite. Finally, the Fe contribution was registered at both spectra, being more intensively detected, as expected, at the surface of the scaffolds obtained by drop-coating methodology (b), given that all the IONPs were concentrated at their surface. Finally, peaks of Na and Cl were detected, which were attributed to the external contributions associated with sample handling.

**Figure 6:**
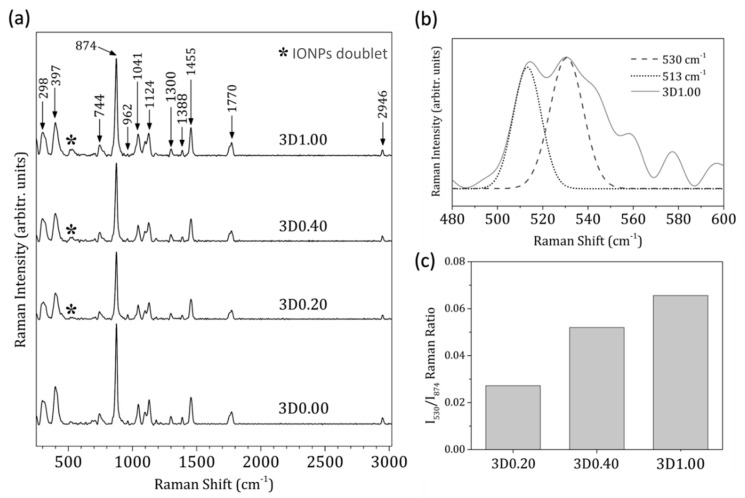
FT-Raman spectra (**a**) of 3D-printed scaffolds with increasing contribution of IONPs from 0.20 to 1.00 mg/scaffold: 3D0.20, 3D0.40, and 3D1.00. FT-Raman spectrum obtained for 3D-printed PLA/HA scaffolds, free of IONPs, with 3D0.00 also included. The main characteristic bands of 3D-printed PLA are assigned, together with the main band of calcium phosphates (962 cm^−1^) and the characteristic doublet assigned to the IONPs (represented by an asterisk). The amplified and deconvoluted doublet for the 3D1.00 scaffold is presented in (**b**). The quantitative evaluation of the FT-Raman spectra with the I530/I874 ratio obtained for the corresponding 3D-printed scaffolds (**c**).

Once the morphological and elemental compositions were evaluated, the main molecular vibrations were analyzed using FT-Raman spectroscopy (Metrohm, Herisau, Switzerland), and the results are presented in Figure 6 for the 3D-printing methodology. The FT-Raman spectrum obtained for the 3D-printed PLA/HA scaffolds, free of IONPs and 3D0.00, fabricated with the same grid pattern and infill density of 100%, was also included as reference material. In this reference FT-Raman spectrum, the corresponding bands attributed to a 3D-printed PLA [24,25] can be observed at 298 cm^−1^ and 397 cm^−1^, assigned to the bending C-O-C and C-CO groups, respectively; at 744 cm^−1^, corresponding to C=O bending; an intense and sharp band at 874 cm^−1^ attributed to C-COO stretching; at 1041 cm^−1^, attributed to skeletal stretching C-CH_3_; at 1124 cm^−1^, attributed to asymmetric rocking CH_3_; at 1300 cm^−1^; attributed to CH bending; at 1388 cm^−1^ and 1455 cm^−1^, both attributed to symmetric bending CH_3_, at 1770 cm^−1^, assigned to C=O asymmetric stretching; and, finally, at 2946 cm^−1^, attributed to CH_3_ symmetric stretching. Moreover, as expected, the band at 962 cm^−1^, attributed to the PO_4_^−3^ symmetric stretching mode of calcium phosphates which corresponds to hydroxyapatite [26], was also detected. Finally, the FT-Raman spectra of the 3D-printed scaffolds with increasing contributions of IONPs, 3D0.20, 3D0.40, and 3D1.00, revealed the corresponding band with the characteristic doublet relative to the iron-oxide nanoparticles (IONPs), amplified and deconvoluted in Figure 6b for the 3D1.00 scaffold, with peaks at 513 cm^−1^ and 530 cm^−1^ attributed to Fe_3_O_4_ [27]. Figure 6c presents the quantitative evaluation of the IONPs in the 3D-printed scaffolds calculated from the FT-Raman spectra by using the ratio of the IONP-related band intensity at 530 cm^−1^ and that of the intense and sharp PLA-related band at 874 cm^−1^ (I530/ I874). As expected, these ratios indicated that the relative contribution of IONPs in the scaffold increased with wt.% of the IONPs incorporated into them, in a linear relationship (R^2^ = 0.9724, equation: y = 0.0192x + 0.0099).

**Figure 7:**
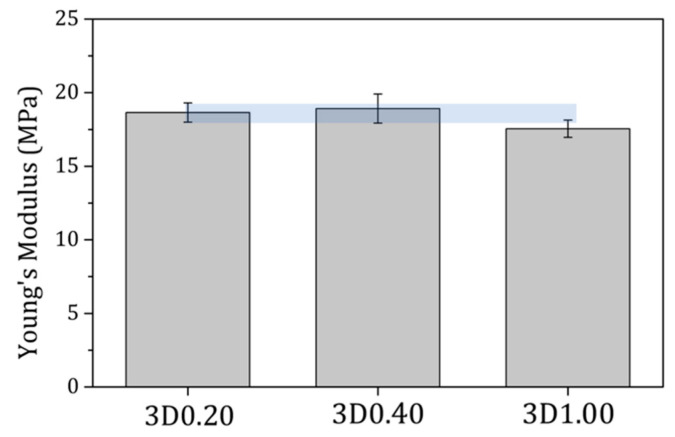
Young’s modulus of the 3D printed scaffolds with increasing contributions of IONPs from 0.20 to 1.00 mg/scaffold: 3D0.20, 3D0.40, and 3D1.00. The bar plot represents mean ± standard error. The blue light color helps to visualize the similar values obtained for the three scaffolds evaluated.

Finally, the mechanical behavior was evaluated by orthogonal compression tests applied on the top surface of the scaffolds in a tensile bench with a 5 kg load cell using a stainless-steel cylinder. The values of Young’s moduli obtained from the stress–strain curves corresponded to a macroscopic evaluation of the samples and are presented in Figure 7 for the scaffolds obtained by the 3D-printing methodology. The results indicated that all three scaffolds with increasing contributions of IONPs had similar values, with non-statistically significant differences of approximately 18 MPa. These values agree with those reported in previous works on 3D-printed PLA-based scaffolds evaluated using similar methodologies [28,29].

### 3.2. AMF Hyperthermia: Experimental Tests

Once characterized, the magnetic-based scaffolds obtained by both methodologies, (1) 3D printing and (2) drop coating, were subjected to the AMF hyperthermia tests. The experimental equipment, based on a planar coil, was first simulated, and validated experimentally, as indicated in Materials and Methods Section 2.4, situating the maximum AFM modulus at the center point of the coil and in its vertical plane (Z-axis), exponentially decreasing with the distance. The optimal distance to situate the scaffolds from the planar coil was within 9.60–24.00 mm. The scaffolds with increasing IONP contribution (wt.%) obtained from both methodologies, the (1) 3D printing and the (2) drop coating, were then, respectively, placed centrally on this Z-axis at a distance from the coil center of 9.65 mm, and subjected to the AMF generator (314 kHz), taking temperature measurements every 10 s (by thermograph images and laser-guided infrared pyrometer) up to 120 s of exposure.

**Figure 8:**
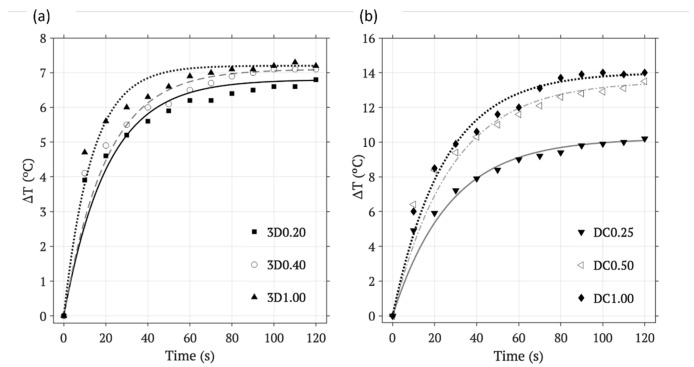
Temperature–time measurements and curves of adjustment for 120 s after AMF exposure on the surface of the scaffolds obtained by (**a**) 3D printing: 3D0.20, 3D0.40, and 3D1.00, and (**b**) drop coating: DC0.25, DC0.50, and DC1.00 at 90 A, 314 kHz, and a coil distance of 9.60 mm.

Figure 8 presents the corresponding temperature–time measurements on the surface of both types of scaffolds for the three compositions tested on each, for an EC intensity of 90 A. The temperature–time measurements (respectively expressed as black/white triangles, circles, squares, rhombus…) obtained for both methodologies included an initial stage during the first 10 s of intense warming, a second stage of a slower gradual heating and a third stage of saturation, where the scaffold temperature remains practically constant. This behavior confirms Newton’s law (Equation (1), already mentioned in the Section 2, for heating processes, which states that the rate of he.at gain in an object is directly proportional to the temperature difference between the object and its surroundings. The hotter an object is, the slower it will heat up; where k is a proportionality constant that depends on the characteristics of the object and environment. Moreover, as expected, the scaffolds with higher concentration in IONPs obtained, for any methodology, required less time of AMF irradiation to achieve the same heating temperature than the lowest concentrated. In the case of the saturation temperature, it was higher for the scaffolds with major concentrations in IONPs, being 7.2 °C above the room temperature for the tested 3D1.00 scaffolds and 13.5 °C for the DC1.00 scaffolds. The results confirmed the efficient incorporation of the IONPs on the scaffolds by the two methodologies and the dependence of the warming achieved with internal parameters of the scaffold, such as the mass of IONPs at the irradiated surface. In fact, the higher warming achieved for the drop-coating methodology for the same conditions and same IONP concentrations is explained by the distribution of the IONPs concentrated at the AMF irradiated surface, instead of the uniform distribution of the same IONP contributions through the entire scaffold, obtained by the 3D-printing methodology. Finally, the temperature of the scaffolds achieved the stationary stage after 120 s of AMF exposure in both methodologies. As already mentioned, the temperature–time measurements obtained for each methodology for each composition were parametrized and the corresponding curves of adjustment were obtained and are represented in Figure 8.This approach allows us to present temperature–AMF exposure time curves that better describe the heating process of the scaffolds before reaching thermal stability. The curves of adjustment obtained for both methodologies, the 3D printing (a) and the drop coating (b), describe the behavior of the data to a high extent, with a R^2^ > 0.99 in all cases.

**Figure 9:**
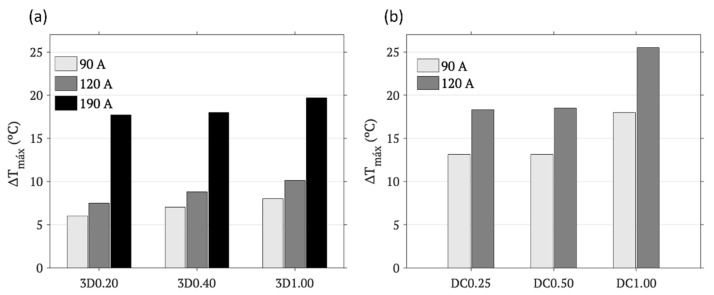
Maximum temperature variation on the surface of the scaffolds obtained by (**a**) 3D printing: 3D0.20, 3D0.40, and 3D1.00, and (**b**) drop coating: DC0.25, DC0.50, and DC1.00 after AMF hyperthermia at different current intensities and a coil distance of 9.60 mm.

Once the temperature–time curves’ dependence on parameters inherent to the scaffold itself, as the contribution in IONPs and their distribution, were evaluated, the influence of external parameters related to the heating system, such as EC intensity and distance from the planar coil, was also experimentally tested for both methodologies. Figure 9 presents the cumulative warming registered at the corresponding surface of the scaffolds, after AMF irradiation for 120 s and a fixed distance of 9.60 mm from the planar coil, for increasing EC intensities from 90 to 120 and/or 190 A. The results for both methodologies, 3D printing (a) and drop coating (b), indicated that the cumulative warming increases with the EC intensity applied, with this parameter able to be modulated to obtain the desired hyperthermia, from moderate (42–50 °C) to ablative (50–55 °C), taking into account that the scaffolds will be at 37 °C in the body. Low EC intensities were proven to be enough to promote moderate hyperthermia for any of the tested 3D-printed scaffolds while ablative hyperthermia will require higher intensities, such as those >120 A. In the case of the drop-coating methodology, both moderate and ablative hyperthermia can be achieved with lower EC intensities, such as those ≤120 A. It is important to notice that during the process, a certain warming occurs at the scaffold itself, without IONP contribution, which contributes to the cumulative warming measures presented.

**Figure 10:**
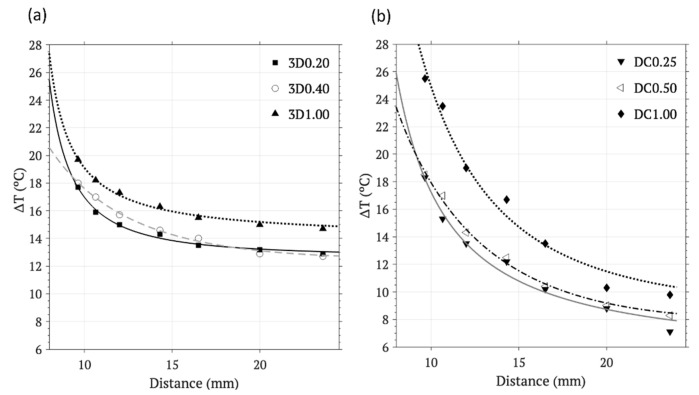
Temperature–distance curves measured after 120 s on the surface of the scaffolds obtained by (**a**) 3D printing: 3D0.20, 3D0.40, and 3D1.00 after AMF hyperthermia at 190 A, and (**b**) drop coating: DC0.25, DC0.50, and DC1.00 after AMF hyperthermia at 120 A.

The influence of the distance from the planar coil, another external parameter, was experimentally tested for both methodologies, and the corresponding temperature–distance curves are shown in Figure 10. As expected, the cumulative warming of the surface of the scaffolds, obtained for both methodologies, decreases with distance from the planar coil in the z axis, and, in addition, it does so by following an inverse quadratic trend in both cases. Adjustment curves are included (R^2^ values > 0.98 in the 3D-printing curves and >0.96 in the drop-coating ones). This result indicates that the warming induced on the IONP-based scaffolds behaves in accordance with the Biot–Savart law, which indicates that the intensity of the magnetic field decreases with the distance squared.

### 3.3. Biological Response In Vitro: Cytotoxicity Assay

**Figure 11:**
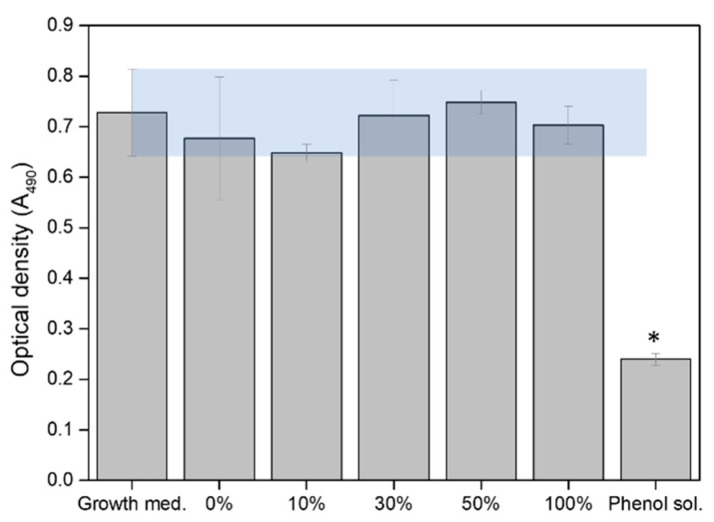
Cell viability detected in osteoblast-like cells (MG63) in the presence of the extracts obtained from 3D-printed scaffolds with 0.40 mg in IONPs (3D0.40), compared to the positive control (phenol sol.) and negative control (cell growth medium) Results are expressed as mean ± standard deviation. Statistical significance was determined to * (*p* ≤ 0.05).

The in vitro evaluation of the cell cytotoxicity caused by the potential release of small particles from the 3D-printed scaffolds in shaking conditions is presented in Figure 11 for the 3D0.40 scaffolds. All the experiments were compared with the negative control of cytotoxicity, which consisted of supplemented cell-culture medium, and the positive control of cytotoxicity, phenol solution. The results revealed optical density values (proportional to metabolic activity) for all the dilutions of the extracts obtained from the scaffold within the range of values obtained for the negative control of cytotoxicity, including the pure extract (100%). The optical density obtained for the negative and positive controls of cytotoxicity validated the experiment, being statistically significantly (*, *p* < 0.05) lower than the viability values obtained for the phenol solution in comparison to the extracts and the negative control.

## 4. Discussion

The present work proposes two new methodologies for the optimal incorporation of IONPs, in mass contribution per scaffold ≤ 1.00 mg, in 3D-printed scaffolds for local hyperthermia therapy applications. The methodologies are as follows: (1) the direct incorporation of the IONPs together with PLA pellets and HA powder in a 3D pellet printer; and (2) the IONPs drop-coated on the already 3D-printed PLA/HA scaffolds were both proven to provide a controlled incorporation of IONPs into the composite scaffolds. Recent works [30,31] have proposed the development of polymeric composite magnetic filaments of magnetite nanoparticles (10, 20 wt.%) and pure PLA, for the same purposes. The authors of those works propose the production of the composite filaments before printing the scaffolds. For this, the process begins with the pulverization of the commercial PLA filament, by cutting it into pieces, grinding the pieces to make granules around 5 mm in diameter, then mixing them with the magnetite nanoparticles, followed by a drying stage and the extrusion of the magnetic composite Fe/PLA filament. In the present work, the direct incorporation of the IONPs (15 nm) with the PLA, as commercial oval pellets with dimensions of 5.0 and 3.5 mm in the major and minor axis, respectively, is demonstrated in a 3D pellet printer, wherein the addition of HA powder (5 wt.%, dimensions around average particle size of 3.29 µm) was also carried out to obtain a magnetic composite scaffold for bone-related applications. Moreover, another methodology, the direct drop coating of the IONPs on the already printed 3D PLA/HA scaffolds, is also proposed, given the versatility that this can offer to the clinician. Both methodologies offer more flexibility for preparing an ad hoc composition according to the specific therapeutic treatment.

Moreover, the present work provides the temperature–time curves obtained for each methodology and their parametrization with the corresponding curves of adjustment obtained, which will allow us to control the hyperthermia therapy applied in a more precise way. The required moderate (43–50 °C) and ablative (50–55 °C) hyperthermia therapy against cancer or resistant infections must be provided with complete control, knowing the kinetics of the process in depth, to apply the required high temperature in the exact tissue/area of interest at the precise time required, by altering the surrounding tissues to the least possible degree. In addition, the influence of parameters such as the distance from the planar coil and the EC intensity were also evaluated in the present work, together with the preliminary cytotoxicity evaluation for an osteoblastic-like cell line. Going into detail, a direct measurement of almost 14 °C of cumulative heating was achieved in the scaffolds obtained for the drop-coating methodology (on the surface where the IONPs were concentrated) after 80 s of AMF application (90 A, 314 kHz, coil distance 9.60 mm), in the case of the DC1.00 scaffolds; 1.00 mg/scaffold. The cumulative heating was 7.2 °C for the methodology based on the 3D direct mixing–printing methodology for the same IONP amount (1.00 mg/scaffold); in this case, it was uniformly distributed through the entire scaffold. These specific data indicated that, as expected, high heating efficiency was obtained for the drop coating, as all of the IONPs are concentrated at the heated surface of the scaffold. In this sense, it is important to notice that the AMF frequency used, and the time of application, agreed with previous published works for the same application [30,31]. In fact, Nain et al. [2] evaluated the dependence of the dissipated power on IONP size and magnetic field frequency (up to 500 kHz), obtaining high specific loss power (SLP) values for the IONPs 15 mm in size, the same as the ones used in the present work, subjected to an AMF at a frequency of 330 kHz, with very close values for higher frequencies. In cases where the intention is to heat the entire scaffold, versus just the external surface heated with the drop-coating methodology, the 3D-printing methodology can be applied. In both cases, the contribution in wt.% of IONPs at the scaffold can be easily increased, in addition to potential variations in the distance from the AFM source and other AMF equipment parameters, in the case that higher hyperthermia levels are required. This work opens new possibilities for the incorporation of magnetic hyperthermia therapy through the personalized 3D-printed tissue-engineered scaffolds. Extensive investigations directly focused on a specific in vivo therapeutic application (anticancer, antibiofilm) must be conducted, including simulations of the tissue heat dissipation.

## 5. Conclusions

The present work demonstrates the ability to obtain multifunctional 3D-printed PLA/HA scaffolds with magnetic properties. The two methodologies proposed to include IONPs, (1) direct 3D printing and (2) drop coating, have been proven to allow for IONP-controlled incorporation, the parametrized thermal behavior of the scaffolds against time/distance (R^2^ > 0.96), and an efficient thermal response to achieve the desired moderate and ablative hyperthermia levels. The influence of the mass of IONPs incorporated and their distribution in the scaffold (uniform distribution: 3D printing; concentrated at the surface: drop coating) was proven, together with the AMF electrical current applied and the working distance. The optimized parameters for the tested conditions confirmed the expected higher heating efficiency of the scaffold surface for the drop-coating methodology, given the higher concentration of IONPs, with a cumulative heating of 14 °C for the scaffolds with 1.00 mg of IONPs after 80 s of AMF exposure. The methodologies proposed allow for the easy personalization of the scaffold composition, microstructure, and shape. Moreover, the parametrization of the thermal results obtained will provide the clinician with the possibility of modulating the conditions and even combining the two methodologies to heat the external surface and/or the entire scaffold when required.

## Figures and Tables

**Table 1:** Technical data of PLA SMARTFIL^®^.

PLA SMARTFIL^®^	Value
Material density	1.24 g/cm^3^
Tensile strength (machine direction)	110 MPa
Tensile strength (transverse direction)	114 MPa
Tensile modulus (machine direction)	3309 MPa
Tensile modulus (transverse direction)	3861 MPa
Heat deflection temperature B	65 °C

**Table 2:** Three-dimensional printing methodology. The weight percentage (wt.%) of PLA, HA, and IONPs incorporated into each scaffold as well as the corresponding labeling of the samples, according to the mass of IONPs (mg) incorporated per scaffold.

3D-Printed Scaffolds	Material			
	PLA	HA	IONPs	
	wt.%	wt.%	wt.%	mg/Scaffold
3D0.20	94.90	5.00	0.10	0.20
3D0.40	94.80	5.00	0.20	0.40
3D1.00	94.50	5.00	0.50	1.00
3D0.00	95.00	5.00	0.00	0.00
PLA	100.00	0.00	0.00	0.00

**Table 3:** Temperature and flow rates used for the 3D printing of the scaffolds.

	*T*_1_ (°C)	*T*_2_ (°C)	Printing Speed (mm/s)	Flow Rate (%)	Infill Density (%)
3D0.20	260	190	19.5	100	100
3D0.40	260	190	18.0	110	
3D1.00	265	190	12.0	110	
3D0.00	250	180	21.0	110	
PLA	210	120	27.0	100	

**Table 4:** Drop-coating methodology. Different masses of IONPs drop-coated on each 3D-printed PLA/HA scaffold, as well as the corresponding labeling of the samples.

Drop-Coated Scaffolds: IONPs mg/Scaffold	0.25	0.50	1.00	0.00
	DC0.25	DC0.50	DC1.00	DC0.00

## Data Availability

The original contributions presented in the study are included in the article; further inquiries can be directed to the corresponding authors.
